# Insights into *Alternanthera mosaic virus* TGB3 Functions: Interactions with *Nicotiana benthamiana* PsbO Correlate with Chloroplast Vesiculation and Veinal Necrosis Caused by TGB3 Over-Expression

**DOI:** 10.3389/fpls.2013.00005

**Published:** 2013-01-31

**Authors:** Chanyong Jang, Eun-Young Seo, Jiryun Nam, Hanhong Bae, Yeong Guk Gim, Hong Gi Kim, In Sook Cho, Zee-Won Lee, Gary R. Bauchan, John Hammond, Hyoun-Sub Lim

**Affiliations:** ^1^Department of Applied Biology, Chungnam National UniversityDaejeon, South Korea; ^2^School of Biotechnology, Yeungnam UniversityGyeongsan, South Korea; ^3^National Institute of Horticultural and Herbal Science, Rural Development AdministrationSuwon, South Korea; ^4^Division of Life Science, Korea Basic Science InstituteDaejeon, South Korea; ^5^Electron and Confocal Microscopy Unit, Beltsville Agricultural Research Center, Agricultural Research Service, United States Department of AgricultureBeltsville, MD, USA; ^6^Floral and Nursery Plants Research Unit, US National Arboretum, Agricultural Research Service, United States Department of AgricultureBeltsville, MD, USA

**Keywords:** AltMV, *potexvirus*, TGB3, chloroplast, PsbO

## Abstract

*Alternanthera mosaic virus* (AltMV) triple gene block 3 (TGB3) protein is involved in viral movement. AltMV TGB3 subcellular localization was previously shown to be distinct from that of *Potato virus X* (PVX) TGB3, and a chloroplast binding domain identified; veinal necrosis and chloroplast vesiculation were observed in *Nicotiana benthamiana* when AltMV TGB3 was over-expressed from PVX. Plants with over-expressed TGB3 showed more lethal damage under dark conditions than under light. Yeast-two-hybrid analysis and bimolecular fluorescence complementation (BiFC) reveal that *Arabidopsis thaliana* PsbO1 has strong interactions with TGB3; *N. benthamiana* PsbO (NbPsbO) also showed obvious interaction signals with TGB3 through BiFC. These results demonstrate an important role for TGB3 in virus cell-to-cell movement and virus-host plant interactions. The Photosystem II oxygen-evolving complex protein PsbO interaction with TGB3 is presumed to have a crucial role in symptom development and lethal damage under dark conditions. In order to further examine interactions between AtPsbO1, NbPsbO, and TGB3, and to identify the binding domain(s) in TGB3 protein, BiFC assays were performed between AtPsbO1 or NbPsbO and various mutants of TGB3. Interactions with C-terminally deleted TGB3 were significantly weaker than those with wild-type TGB3, and both N-terminally deleted TGB3 and a TGB3 mutant previously shown to lose chloroplast interactions failed to interact detectably with PsbO in BiFC. To gain additional information about TGB3 interactions in AltMV-susceptible plants, we cloned 12 natural AltMV TGB3 sequence variants into a PVX expression vector to examine differences in symptom development in *N. benthamiana*. Symptom differences were observed on PVX over-expression, with all AltMV TGB3 variants showing more severe symptoms than the WT PVX control, but without obvious correlation to sequence differences.

## Introduction

The chloroplast has for many years been recognized as susceptible to damage during plant virus infections. Infection with *Tobacco mosaic virus* (TMV) was shown to interfere with starch mobilization in local lesions on tobacco (Holmes, 1931). Later, TMV particles were observed in association with chloroplasts (Esau and Cronshaw, 1967; Granett and Shalla, 1970), while Reinero and Beachy (1986) showed that TMV coat protein (CP) accumulated within chloroplasts of infected tobacco. Schoelz and Zaitlin (1989) demonstrated that TMV genomic RNA, but not subgenomic RNA, enters tobacco chloroplasts, and suggested that the CP detected may be translated by chloroplast ribosomes from the genomic RNA due to the presence of a Shine–Dalgarno sequence upstream of the CP initiation codon.

Other viruses have also been shown to associate with chloroplasts. Both CP and the HC-Pro of *Potato virus Y* (PVY) were detected in chloroplasts (Gunasinghe and Berger, 1991). The 6K2 protein of the potyvirus *Turnip mosaic virus* (TuMV) is an integral membrane protein that first forms vesicles at the endoplasmic reticulum (ER), and then traffics to the periphery of the chloroplasts, where large invaginations appear to result from chloroplast extrusions engulfing aggregated vesicles (Wei et al., 2010). The Triple Gene Block (TGB) 2 (TGB2) protein of *Barley stripe mosaic virus* also accumulates in chloroplasts (Torrance et al., 2006), as does the CP of *Cucumber necrosis virus* (Xiang et al., 2006). We have recently shown that the CP of *Lolium latent virus* (LoLV) has a chloroplast transit peptide, and that blocking cleavage of the transit peptide has a dramatic effect on LoLV systemic movement (Vaira et al., 2012).

*Turnip yellow mosaic virus* (TYMV) has been known for many years to induce vesicles in the exterior of the chloroplasts (Chalcroft and Matthews, 1966), which were assumed to be the site of viral RNA synthesis (Ushiyama and Matthews, 1970), and have more recently been demonstrated to harbor the TYMV replicase proteins (Prod’Homme et al., 2001, 2003). Cells in areas of white (severe) mosaic were observed to have enlarged chloroplasts with numerous vesicles, including more very large vesicles than in yellow-green areas; disintegrated chloroplasts were also observed in such areas (Chalcroft and Matthews, 1966).

A number of studies have identified responses of Photosystem II (PS II) to infection with various viruses, including differential effects on components of the Oxygen-evolving complex (OEC); Abbink et al. (2002) utilized the yeast two-hybrid (Y2H) system to show that the RNA helicase domain of the 126-kDa replicase of TMV-U1 (but not of TMV-Ob) interacted with the 33-kDa subunit of the OEC, also known as PsbO. Virus-Induced Gene Silencing (VIGS) of *Nicotiana benthamiana psbO* (NbPsbO) with the *Tobacco rattle virus* (TRV) system resulted in a 10-fold increase in TMV accumulation, and also increased accumulation of *Potato virus X* (PVX; *Potexvirus*) and *Alfalfa mosaic virus* several-fold; inhibition of PS II with the herbicide [3-(3,4-Dichlorophenyl)-1,1-dimethyurea] (DCMU) also increased accumulation of TMV (Abbink et al., 2002). Other tobamoviruses and *Cucumber mosaic virus* have been reported to differentially affect components of PS II, reducing the levels of the 24-kDa (PsbP) and 16-kDa (PsbQ) subunits but not PsbO (Takahashi et al., 1991; Takahashi and Ehara, 1992; Rahoutei et al., 2000; Pérez-Bueno et al., 2004; Sui et al., 2006).

Specific interactions of other virus proteins with chloroplast proteins include those observed between the CP of PVX and the chloroplast transit peptide of plastocyanin; VIGS of plastocyanin using the TRV system reduced both the severity of PVX symptoms and the accumulation of PVX CP (Qiao et al., 2009). Chloroplast phosphoglycerate kinase (cPGK) was found to interact with the 3′-untranslated region (UTR) of *Bamboo mosaic virus* (BaMV), another member of the genus *Potexvirus*; when cPGK was silenced by VIGS with TRV, accumulation of BaMV CP was also reduced, suggesting that the replication site of BaMV is associated with the chloroplast and that cPGK may target the RNA to the chloroplast membrane (Lin et al., 2007). *Tomato mosaic virus* CP interacts with a thylakoid membrane protein IP-L (Zhang et al., 2008), while PVY (*Potyvirus*) CP has been shown to interact with the large subunit of Rubisco (Feki et al., 2005), and PVY HC-Pro with the domain of the nuclear-encoded chloroplast-division related protein MinD required for dimerization (Jin et al., 2007). TuMV CP interacts with an otherwise unidentified 37 kDa chloroplast protein (McClintock et al., 1998). HC-Pro of another potyvirus, *Sugarcane mosaic virus*, interacts in the cytoplasm with the chloroplast transit peptide of maize ferredoxin-5 (Fd V), possibly disturbing chloroplast import of mature Fd V (Cheng et al., 2008). The replication-associated CI protein of *Plum pox virus* (PPV) interacts with nuclear-encoded photosystem I (PS I) protein PSI-K of *N. benthamiana*, and down-regulation of the gene encoding PSI-K increased PPV accumulation; PPV infection itself results in reduced levels of PSI-K protein (Jiménez et al., 2006). The P1 protein of *Soybean mosaic virus* interacts with both the chloroplast transit peptide and the mature nuclear-encoded chloroplast Rieske Fe/S protein of several host species, but only weakly with the corresponding protein of the non-host *Arabidopsis thaliana* (Shi et al., 2007).

We have previously shown that the triple gene block 3 (TGB3) of the potexvirus *Alternanthera mosaic virus* (AltMV) localizes to the chloroplast, whereas TGB3 of PVX, the type member of the genus *Potexvirus*, accumulates at the ER (Lim et al., 2010a). We also used deletion mutants and site-directed mutagenesis to demonstrate that chloroplast localization is due to a signal in the N-terminal domain, and that mutation of VL(17,18)AR in the N-terminal domain was sufficient to both prevent chloroplast localization, and to severely limit virus movement to a few cells within the epidermal layer (Lim et al., 2010a). Over-expression of AltMV TGB3 as an additional gene from either AltMV or from a PVX vector resulted in veinal-associated necrosis, chloroplast malformation and vesicular invaginations of chloroplast membranes, and cytoplasmic membrane proliferation (Lim et al., 2010a). Fluorescence *in situ* hybridization showed that AltMV RNA was closely associated with the chloroplasts, which combined with chloroplast invagination when TGB3 was over-expressed suggests that the chloroplast is the site of AltMV replication (Lim et al., 2010a). In related preliminary work, we have shown that AltMV TGB1 interacts with chloroplast β-ATPase of both *A. thaliana* and *N. benthamiana* (Nam et al., 2012).

Here we examine the interactions of AltMV TGB3 with host PS II OEC protein PsbO of both *A. thaliana* and *N. benthamiana*, using deletion mutants to determine the TGB3 domains involved in the interactions, and demonstrate significant chloroplast damage when plants over-expressing TGB3 are grown under dark conditions. We have also examined over-expression of a series of natural AltMV TGB3 sequence variants, and determined that all variants induced chloroplast damage under dark conditions.

## Materials and Methods

### Virus isolates, cDNA clones, and plant maintenance

*Alternanthera mosaic virus* 3–7 (AltMV 3–7) is derived from an infectious clone prepared from *Phlox stolonifera* isolate AltMV-SP, and was used for all experiments unless otherwise noted; AltMV 3–1 and AltMV 4–7 are also derived from AltMV-SP, and share an identical TGB3 amino acid sequence (Lim et al., 2010b). *P. stolonifera* isolates AltMV-BR and AltMV-PA, and *Portulaca grandiflora* isolate AltMV-Po have been described previously (Hammond et al., 2006a,b). Complementary DNA clones of the 3′-terminal region of AltMV-PGL (from *P. carolina*), AltMV-PLR (from hybrid annual phlox), AltMV-Po57 (from *P. grandiflora*), AltMV-NAN (from *Nandina domestica*), and AltMV-CIN (from hybrid *Pericallis*) were produced and sequenced essentially as reported for AltMV-BR and AltMV-Po (Hammond et al., 2006a,b). The TGB3 of all isolates except AltMV-MU (see below) were amplified using primers *Xho*I-F-TGB3 and *Bam*HI-R-TGB3 (Table 1). The full sequence of European portulaca isolate AltMV-MU was reported by Ivanov et al. (2011), and the amino acid sequence of AltMV-MU TGB3 was derived by polymerase chain reaction (PCR) from an AltMV-Po template using *Xho*I-F-TGB3 paired with a reverse primer *Bam*HI-R-TGB3-MU (Table 1) to introduce the substitution R62K which differentiates the TGB3 of these isolates. The *Xho*I and *Bam*HI sites were introduced to allow cloning of the PCR products into pGD, pGDG, or pGDR (Goodin et al., 2002) for transient expression by agroinfiltration. The sequences of all TGB3 constructs were verified by sequencing.

**Table 1:** **Primers used in this study**.

Clone	5′-Oligo	5′-Oligo sequence	3′-Oligo	3′-Oligo sequence	Feature
**PRIMERS USED IN BINARY VECTORS CONSTRUCTS FOR *AGROBACTERIUM* INFILTRATION EXPERIMENTS**					
AltMV TGB3	*Xho*I-F-TGB3	CTCGAGAAATGCCCTATCTTGTAGAG	*Bam*HI-R-TGB3	CAAACGGATCCTAAAACCTAAGCCCG	*Xho*I and *Bam*HI
AltMV TGB3 MU	*Xho*I-F-TGB3	CTCGAGAAATGCCCTATCTTGTAGAG	*Bam*HI-R-TGB3-MU	CAAACGGATCCTAAAACTTAAGCCCG	*Xho*I and *Bam*HI
NbPsbOI	*Xho*I-F-NbPsboI	GAGCTCGAGAAATGGCTGCCTCTCTACAAGCAGCTG	*Kpn*I-R-NbPsboI	GAGGGTACCCCCTTCAAGTTGGGCATACCAGATACC	*Xho*I and *Kpn*I
**PRIMERS USED FOR YEAST-TWO-HYBRID EXPRESSION OF AltMV TGB3**					
pGBKT7-TGB3	*Eco*RI F	GAGAGAATTCATGCCCTATCTTGTAGAG	*Bam*HI R	GAGAGGATCCCTAAAACCTAAGCCAAAGCA	*Eco*RI and *Bam*HI
**AltMV TGB3 CONSTRUCTS IN BiFC VECTORS**					
BiFC: AltMV TGB3	*Xho*I-F-TGB3 BiFC	GAGCTCGAGATGCCCTATCTTGTAGAGGCGGCC	*Xma*I-R-TGB3 BiFC	GAGCCCGGGCCCAAACCTAAGCCCGGTTAAATAGTCTCC	*Xho*I and *Xma*I
BiFC: AltMV TGB3-ΔN15	*Xho*I-F- TGB3-ΔN15 BiFC	GAGCTCGAGATGGTCCTTGCTGCTCTTAGGCCA	*Xma*I-R-TGB3 BiFC	GAGCCCGGGCCCAAACCTAAGCCCGGTTAAATAGTCTCC	*Xho*I and *Xma*I
BiFC: AltMV TGB3-ΔC15	*Xho*I-F-TGB3 BiFC	GAGCTCGAGATGCCCTATCTTGTAGAGGCGGCC	*Xma*I-R-TGB3-ΔC15 BiFC	GAGCCCGGGCCCTGGTGCGACGGGTCCACAATCT	*Xho*I and *Xma*I
**PsbOI CONSTRUCTS IN BiFC VECTORS**					
BiFC: AtPsbOI	*Xho*I-F-AtPsboI BiFC	GAGCTCGAGATGGCAGCCTCTCTCCAATCCACC	*Xma*I-R-AtPsboI BiFC	GAGCCCGGGCCCCTCAAGTTGACCATACCAC	*Xho*I and *Xma*I
BiFC: NbPsbOI	*Spe*I-F-NbPsboI BiFC	GAGACTAGTATGGCTGCCTCTCTACAAGC	*Xho*I-R-NbPsboI BiFC	GAGCTCGAGCCCTTCAAGTTGGGCATACC	*Spe*I and *Xho*I
**TGB3 OVER-EXPRESSION EXPERIMENTS**					
PVX(AltMV TGB3+)	*Bam*HI-F-TGB3	GAGAGGATCCATGCCCTATCTTGTAGAG	*Mlu*I-R-TGB3	GAGAACGCGTCTACCTGATGGTCTCTGGTGCG	*Bam*HI and *Mlu*I
PVX(AltMV TGB3 MU+)	*Bam*HI-F-TGB3	GAGAGGATCCATGCCCTATCTTGTAGAG	*Mlu*I-R-TGB3-MU	GAGAACGCGTCTACTTGATGGTCTCTGGTGCG	*Bam*HI and *Mlu*I

Triple gene block 3 sequences of the various AltMV isolates were also separately introduced into infectious clone PVX-MCS as an additional gene, essentially as described (Lim et al., 2010a), forming PVX(TGB3 AltMV+) variants. Infectious RNA transcripts of wild-type (WT) PVX and the PVX(TGB3 AltMV+) variants were transcribed *in vitro* after linearization with *Spe*I, and inoculated to young plants of *N. benthamiana* as described (Petty et al., 1989; Lim et al., 2010a). Seven days post inoculation (dpi) plants inoculated with AltMV 4–7, WT PVX, or PVX(TGB3 AltMV+) variants were separately incubated under either light (16 h light/8 h dark) or continuous dark conditions at 16°C.

*Alternanthera mosaic virus* was maintained by mechanical inoculation on *N. benthamiana* using 1% K_2_HPO_4_ and carborundum powder as an abrasive. Plants were grown in 10 cm pots in an insect-proof greenhouse at 25°C, under a 14-h light regime. Plants of *A. thaliana* and *N. benthamiana* for agroinfiltration were grown under similar conditions, and were fully imbibed by standing pots in water for 4–5 h prior to agroinfiltration.

### Yeast two-hybrid assays

*Alternanthera mosaic virus* 3–7 TGB3 was subcloned into pGBKT7 at *Eco*RI and *Bam*HI sites, in fusion with the Gal4 DNA-BD, and the resulting plasmid (pGBKT-TGB3) was used to transform yeast competent cells (strain AH109) for bait protein expression (Becker et al., 1991). *A. thaliana* cDNA library (CD4–30, Arabidopsis Biological Resource Center, www.abrc.osu.edu) in pAD-GAL4-2.1 (in fusion with Gal4 DNA-AD) plasmid was used to transform yeast competent cells containing pGBKT-TGB3. Transformant cells were screened on SD agar His-, Leu-, Trp- plus Aureobasidin A. Yeast colonies obtained were grown on the same media including the chromogenic substrate X-α-galactosidase; only colonies developing blue color were considered positive. *A. thaliana* genes encoding proteins identified as binding TGB3 were amplified from the selected yeast colonies using appropriate primers, sequenced, and identified by BLAST analysis against the NCBI database (Lim et al., unpublished data).

*Arabidopsis thaliana* PsbO1 gene (oxygen-evolving enhancer protein 1-1, TAIR: AT5G66570, Acc. No. NM126055) and the homologous gene in *N. benthamiana* (NbPsbO; Acc. No. AY952375; Sui et al., 2006) were examined in this study. The corresponding NbPsbO gene (about 1000 bp), was amplified from *N. benthamiana* total RNA using primers *Xho*I-F-NbPsboI and *Kpn*I-R-NbPsboI (Table 1) based on the NbPsbO sequence, following cDNA synthesis with a polyT primer, sequenced, and fused to the C-terminus of the *Discosoma* sp. red fluorescent protein (DsRed) in pGDR for agroinfiltration. NbPsbO was also cloned into pGDG as a fusion to the C-terminus of the Green Fluorescent Protein (GFP), and pSPYCE/pSPYNE vector variants (Waadt et al., 2008) for subcellular localization and bimolecular fluorescence complementation (BiFC) assays, respectively.

The insertion gene fragment of NbPsbO for BiFC was derived from PCR using cDNA of *N. benthamiana* which was synthesized from *N. benthamiana* total mRNA. Primers *Spe*I-F-NbPSBOI BiFC and *Xho*I-R-NbPSBOI BiFC (Table 1) were used to amplify the 999-bp PCR product of NbPsbO with oligo(dT)-primed *N. benthamiana* cDNA as template. The primer set were synthesized based on the sequence of *N. benthamiana* chloroplast photosynthetic oxygen-evolving protein 33 kDa subunit (PsbO) mRNA (GenBank ID: AY952375.1; Sui et al., 2006).

### Bimolecular fluorescence complementation assays

The pSPYCE(M), pSPYCE(MR), pSPYNE173, and pSPYNE(R)173 vectors (Waadt et al., 2008) were used for insertion of TGB3 variants, *A. thaliana* PsbBO1 (AtPsbO1), and NbPsbO, as fusions with the C-terminal (SPYCE constructs) and N-terminal (SPYNE constructs) domains of the enhanced Yellow Fluorescent Protein (eYFP), respectively, using the primers shown in Table 1. Binary plasmids were transformed into *Agrobacterium tumefaciens* strain EHA105 by standard protocols (Johansen and Carrington, 2001), and agroinfiltrated in each combination of TGB3 and PsbO, as well as homologous TGB3 combinations. Transient expression in *N. benthamiana* was performed by agroinfiltration (at OD_600_ = 0.6) with each pSPYCE and pSPYNE variant; pGD-p19 (Bragg and Jackson, 2004) was included at 1:10 ratio in all infiltrations as described (Lim et al., 2009). AtPsbO1 combinations were examined by agroinfiltration of *A. thaliana*, and all other combinations by agroinfiltration of *N. benthamiana*. For all combinations, eYFP fluorescence was observed at 3 days post-agroinfiltration (dpa) by laser scanning confocal microscopy (LSCM; see below).

### Interaction of GFP:PsbO and DsRed:TGB3 fusions

*Nicotiana benthamiana PsbO* was fused to the C-terminus of GFP, and TGB3 to the C-terminus of DsRed, in the vectors pGDG and pGDR (Goodin et al., 2002), respectively. Transient expression by agroinfiltration of *N. benthamiana* was performed as for BiFC assays, and fluorescent protein localization and interactions observed by LSCM (see below) at 3 dpa.

### Detection of fluorescent protein expression in *N. benthamiana*

Laser scanning confocal microscopy using a Zeiss LSM 710 microscope was used for detection of GFP, DsRed, and chloroplast autofluorescence as described by Lim et al. (2010a). For BiFC, eYFP was excited at 514 nm (Argon laser, MBS458/514 filter set) and the emission detected at 514–550 nm. When required, nuclei were stained with 4′-6-diamidino-2-phenylindole dihydrochloride (DAPI) essentially as described by Deng et al. (2007). DAPI fluorescence was excited with a 405-nm laser, with emission detected at 410–475 nm.

Zeiss Zen™ 2009 software was used to obtain images with maximum intensity projection (MIP) of *Z*-stacks (1 μm slices, 2–80 focal planes) of leaves from the top of the epidermis into the mesophyll, or within the mesophyll.

### Electron microscopy

Tissue samples (ca. 2 mm × 1 mm) were excised from leaves of *N. benthamiana* infected with WT PVX, PVX over-expressing AltMV TGB3 or AltMV 3–1 over-expressing TGB3 (Lim et al., 2010a), and processed for embedding according to Lawson and Hearon (1973). Ultrathin sections were examined with a JEOL 100CX II transmission electron microscope (JEOL Ltd.) equipped with an AMT HR digital camera system (Advanced Microscopy Techniques Corp.).

## Results

### Yeast two-hybrid interactions

Several proteins were identified by screening of an *Arabidopsis* cDNA library with AltMV TGB3 as bait; because the PS II OEC protein, AtPsbO1, showed the strongest interaction (data not shown), and because we had previously shown that TGB3 localizes to the chloroplast (Lim et al., 2010a), we selected PsbO for detailed examination. The interactions detected by screening of the *Arabidopsis* cDNA library were confirmed using the full-length AtPsbBO1 and NbPsbO proteins for BiFC.

### Bimolecular fluorescence complementation assays

Reciprocal interactions between AltMV TGB3 and AtPsbO1 were detected by BiFC only when both constructs were expressed with the eYFP fragment fused to the N-terminus of the test protein. No interaction was observed when either TGB3 or PsbO1 was fused upstream of the eYFP fragment, and no homologous TGB3 interaction was observed in any combination (Table 2).

**Table 2:** **Interactions of AltMV TGB3 and AtPsbO1 as detected by BiFC**.

Combination	Interaction
TGB3-eYFP_C155_/AtPsbO1-eYFP_N173_	−
TGB3-eYFP_N173_/AtPsbO1-eYFP_C155_	−
eYFP_C155_-TGB3/eYFP_N173_-AtPsbO1	+
eYFP_N173_-TGB3/eYFP_C155_-AtPsbO1	+
TGB3-eYFP_C155_/eYFP_N173_-AtPsbO1	−
TGB3-eYFP_N173_/eYFP_C155_-AtPsbO1	−
eYFP_C155_-TGB3/AtPsbO1-eYFP_N173_	−
eYFP_N173_-TGB3/AtPsbO1-eYFP_C155_	−
TGB3-eYFP_C155_/TGB3-eYFP_N173_	−
eYFP_N173_-TGB3/eYFP_N173_-TGB3	−
eYFP_C155_-TGB3/TGB3-eYFP_N173_	−
TGB3-eYFP_N173_/TGB3-eYFP_C155_	−

**Figure 1:**
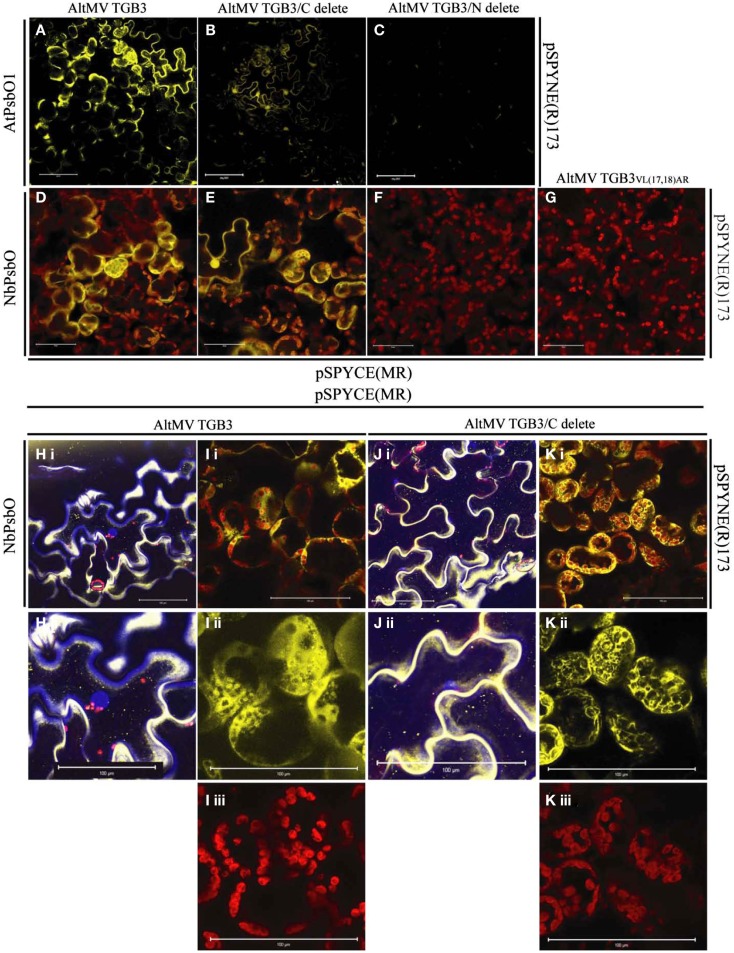
**BiFC interactions between AtPsbO1 or NbPsbO and variants of AltMV TGB3**. **(A–C)** Reactions of AtPsbO1 with TGB3 in epidermal cells of *A. thaliana*. **(D–K)** Reactions of NbPsbO with TGB3 in mesophyll cells of *N. benthamiana*. **(A,D,H,I)** Full-length TGB3. **(B,E,J,K)** C-terminal deleted TGB3. **(C,F)** N-terminal deleted TGB3. **(G)** TGB3_VL(17,18)AR_. **(H)**, **(i)** Full-length TGB3 in epidermal cells, with nuclei revealed by DAPI staining; and **(ii)** enlargement of central area of **(i)**, showing BiFC interaction along cell wall, and absence of interaction associated with central nucleus (blue). **(I)** Full-length TGB3 in mesophyll cells, showing **(i)**, BiFC interaction around chloroplasts; **(ii)**, BiFC (yellow) channel image of enlarged area of **(i)** showing punctate interaction on some chloroplasts, interaction surrounding envelope of other chloroplasts, and in cytoplasm around chloroplasts; **(iii)**, Red channel, showing chloroplast autofluorescence of same enlarged area as **(ii)**. **(J)**, **(i)** C-terminal deleted TGB3 in epidermal cells, with nuclei revealed by DAPI staining; and **(ii)** enlargement of lower left area of **(i)**, showing nucleus (blue) appressed to cell wall with absence of nuclear-associated BiFC interaction. **(K)** C-terminal deleted TGB3 in mesophyll cells, showing **(i)** BiFC interaction around chloroplasts; **(ii)**, BiFC (yellow) channel image of enlarged area of **(i)**, showing interaction at chloroplast envelope as well as in cytoplasm around chloroplasts; **(iii)**, Red channel (chloroplast autofluorescence) of same enlarged area as **(ii)**. Note BiFC reaction with full-length and C-terminal deleted TGB3, and absence of interaction with N-terminal deleted TGB3 or TGB3_VL(17,18)AR_. AtPsbO1 and NbPsbO were fused with the N-terminal domain of YFP in pSPYNE(R)173, while TGB3 variants were fused to the C-terminal domain of YFP in pSPYCE(MR). Red is chloroplast autofluorescence from mesophyll cells. Bars = 100 μm.

**Figure 2:**
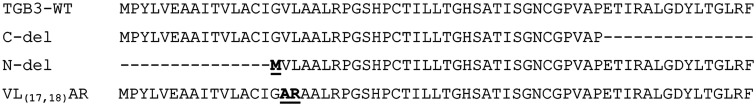
**Amino acid sequence of mutants of AltMV TGB3 used for BiFC reactions**. Altered residues are shown in bold and underlined.

Similar interactions were observed between AltMV TGB3 and both AtPsbO1 and NbPsbO, in *A. thaliana* and *N. benthamiana*, respectively, in both epidermal and mesophyll layers (Figures 1A,D,H,I; and data not shown). As we had previously demonstrated that N-terminal TGB3 deletions of as much as 16 residues, and C-terminal deletions of at least 11 residues were still directed to the chloroplast (Lim et al., 2010a), we utilized N-terminal and C-terminal mutants of TGB3 (Figure 2) in BiFC experiments to determine which domains of TGB3 were responsible for interaction with both AtPsbBO1 and NbPsbO. In each case, deletion of the C-terminal 15 residues reduced but did not eliminate the interaction (Figures 1A,E,J,K), whereas deletion of N-terminal residues 2–16 essentially eliminated the interaction with either PsbO (Figures 1C,F).

The interactions of PsbO and either TGB3 (Figures 1D,I) or C-terminally deleted TGB3 (Figures 1E,K) were clearly localized around the chloroplasts in the mesophyll layer, with additional fluorescence at the periphery of the cell. The intimacy of the association with chloroplasts varied between cells; in some cells the BiFC interaction was clearly localized to the chloroplast envelope, as well as in the cytoplasm surrounding the chloroplasts [Figures 1Iii, Kii]. Interaction with some chloroplasts showed a more punctate appearance [Figure 1Iii] similar to that previously observed with GFP:TGB3 and DsRed:TGB3 fusions (Lim et al., 2010a), but other chloroplasts in the same cell appeared to be surrounded by the interaction as seen with C-terminal fusions of GFP and DsRed to full-length TGB3, TGB3ΔN9, and TGB3ΔN16ΔC11 (Lim et al., 2010a). Interestingly, epidermal cells were also labeled by eYFP_C155_-TGB3/eYFP_N173_-PsbO interactions (Figures 1A,B,D,E,H,J) although GFP:TGB3 and DsRed:TGB3 fusions were previously found to be essentially absent from epidermal tissue (Lim et al., 2010a).

In the epidermal cells, the distribution of the BiFC signal was more dispersed at the periphery of the cells (Figures 1A,E,H,J). Although globular accretions of BiFC signal observed in epidermal cells (Figures 1B,E) appear to be nuclei, no association of BiFC signal with nuclei could be identified in leaf pieces infiltrated with DAPI; epidermal aggregations of BiFC signal were instead observed primarily in curves of the cell wall (Figures 1H,J). These epidermal aggregates of eYFP_N173_-PsbO and eYFP_C155_-TGB3 were therefore presumed to result from over-expression, rather than to reflect a specific association with a cellular component, and no nuclear association could be confirmed. Little PsbO would normally be expected in the epidermal layer, due to the low frequency of chloroplasts in this tissue, and no nuclear association of TGB3 has been identified.

Overall, these results suggested that it is the N-terminal domain of TGB3 which interacts with PsbO. As we have previously demonstrated that the region between residues 16 and 20 is critical for chloroplast targeting, and that mutation VL(17,18)AR (Figure 2) ablates direct chloroplast interaction (Lim et al., 2010a), we next examined the interaction of TGB3_VL(17,18)AR_ with PsbO. We hoped to determine whether chloroplast localization is a prerequisite for interaction with PsbO, or alternatively, whether interaction with PsbO is required for chloroplast localization of TGB3. No interaction was observed between NbPsbO and TGB3_VL(17,18)AR_ in *N. benthamiana* (Figure 1G), suggesting that these TGB3 residues (or at least L18; see below) are critical for the interaction with PsbO as well as chloroplast localization.

### Interaction of GFP:PsbO and DsRed:TGB3 fusions

**Figure 3:**
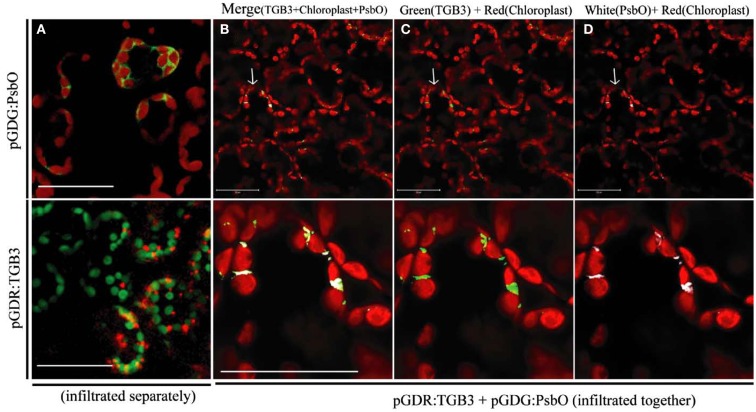
**Interaction between GFP:PsbO (NbPsbO) and DsRed:TGB3 in mesophyll tissue of *Nicotiana benthamiana***. Constructs pGDG:PsbO and pGDR:TGB3 were infiltrated separately **(A)**, or co-agroinfiltrated **(B–D)** into leaves of *N. benthamiana* and examined by LSCM at 3 dpa. **(A)** Upper, GFP:PsbO (shown as green) expressed alone, showing localization around the chloroplasts, with chloroplast autofluorescence shown in red; lower, DsRed:TGB3 (shown as red) showing punctate spots associated with chloroplasts, with chloroplast autofluorescence shown in green. **(B–D)** The upper row shows multiple cells with typical morphology of the mesophyll layer, and red chloroplast autofluorescence. The lower row shows a magnified image of the area indicated by an arrow in the upper panel. **(B)** Merge of DsRed:TGB3 (shown as green) with GFP:PsbO (shown as white) and chloroplast autofluorescence (red). **(C)** DsRed:TGB3 (green) and chloroplast autofluorescence (red). **(D)** GFP:PsbO (white) and chloroplast autofluorescence (red). Note co-localization of DsRedD:TGB3 and GFP:PsbO at areas where chloroplasts appear to be in close contact. Scale bar = 50 μm.

GFP:PsbO (NbPsbO) expressed by agroinfiltration of *N. benthamiana* (with pGDG:PsbO) in the absence of DsRed:TGB3 localized around the chloroplasts of mesophyll cells (Figure 3A, upper), whereas DsRed:TGB3 (from pGDR:TGB3) localized to the chloroplasts as punctate spots (Figure 3A, lower). When GFP:PsbO and DsRed:TGB3 were co-expressed, almost complete co-localization was observed in mesophyll cells, apparently at points where two chloroplasts were in close contact (Figures 3B–D), confirming the interactions visualized by BiFC. The GFP:PsbO/DsRed:TGB3 interaction was predominantly punctate, by comparison to the BiFC interaction, which displayed a mix of punctate spots at the chloroplast surface, distribution surrounding the chloroplast, and in the adjacent cytoplasm and cell periphery (Figure 1). This suggests that localization of TGB3 predominates in the GFP:PsbO/DsRed:TGB3 interaction, whereas PsbO localization may be dominant in the BiFC interaction.

### Electron microscopy

**Figure 4:**
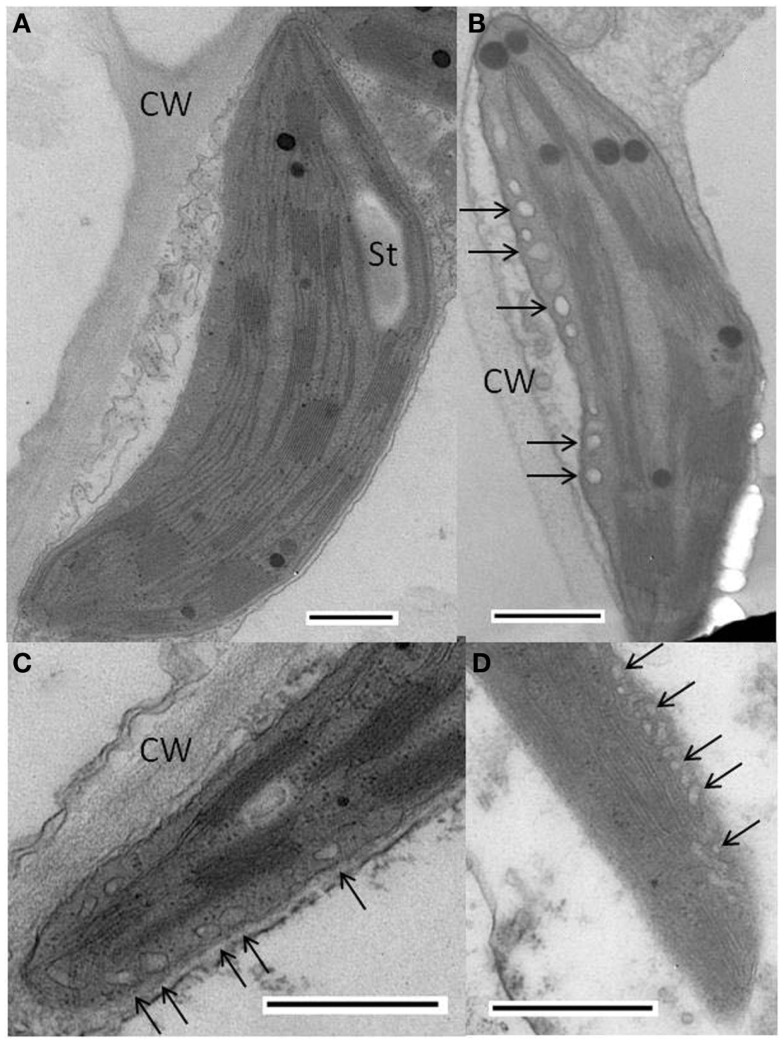
**Cytopathology associated with AltMV TGB3 over-expression**. **(A)** Normal chloroplast in thin section of *N. benthamiana* infected with PVX. **(B,C)** Vesicles (arrows) observed in chloroplast of *N. benthamiana* infected with PVX over-expressing AltMV TGB3 as an added gene. **(D)** Vesicles (arrows) in chloroplast of *N. benthamiana* infected with AltMV over-expressing AltMV TGB3 as an added gene. CW, cell wall; St, starch. Scale bars in each panel = 500 nm.

**Figure 5:**
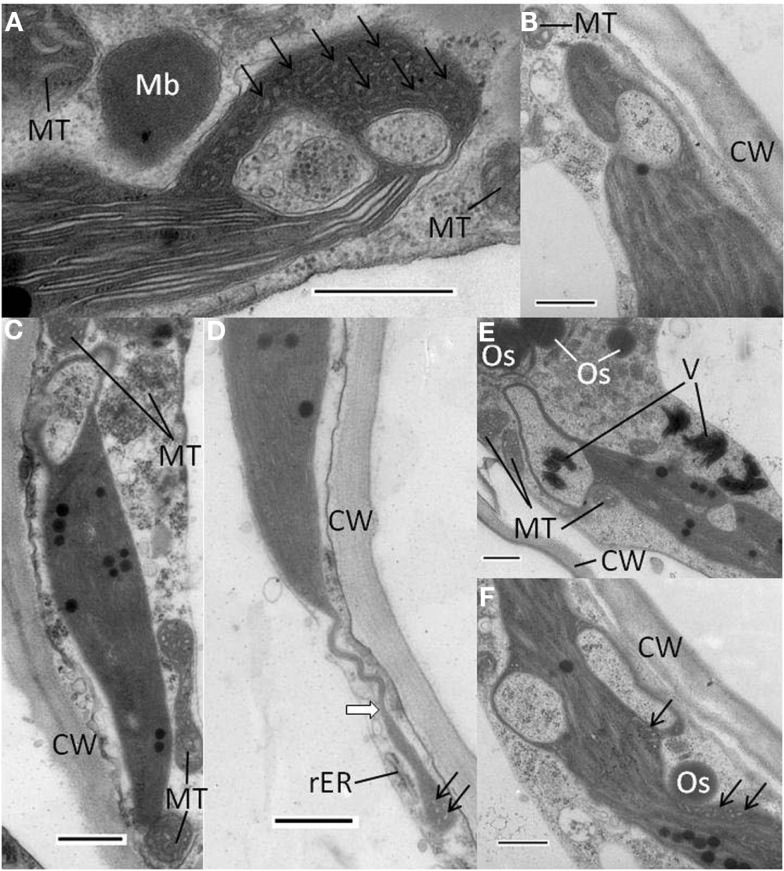
**Abnormal chloroplast morphology observed in *N. benthamiana* infected with AltMV over-expressing TGB3 as an added gene**. **(A)** Large invagination and multiple vesicles present at end of a chloroplast, with an adjacent paracrystalline microbody that may be a peroxisome. **(B)** Large invagination open to cytoplasm near one end of a chloroplast. **(C)** Large apparent cytoplasmic invagination at one end of a chloroplast. **(D)** Stromule-like extension (white arrow) from the end of a chloroplast. **(E)** Two large invaginations of cytoplasmic material into a chloroplast. Note apparent virion aggregates inside apparently fully enclosed area, and larger virion aggregates near invagination open to cytoplasm. **(F)** Chloroplast with two large apparently closed cytoplasmic invaginations, and multiple small vesicles in constricted region adjacent to possible TGB1 aggregate. CW, cell wall; Mb, microbody or peroxisome; MT, mitochondrion; Os, osmiophilic globule or plastoglobule; rER, rough endoplasmic reticulum; V, virion aggregates: arrows indicate areas of small vesicles. Scale bars in each panel = 500 nm.

Plants infected with WT PVX had essentially normal chloroplasts (Figure 4A), whereas in plants infected with PVX over-expressing AltMV TGB3, abnormal chloroplasts with approximately spherical vesicular invaginations at the peripheral membrane could frequently be found (Figures 4B,C). Similar vesicles were also observed in chloroplasts of plants infected with AltMV over-expressing TGB3 (Figure 4D), as could some much larger vesicles (Figure 5).

Additional types of abnormal chloroplasts were frequently observed in plants infected with AltMV over-expressing TGB3. Significant invaginations of cytoplasmic material were found, typically toward the ends of chloroplasts (Figures 5A–C,E), and in some instances combined with significant quantities of irregular small vesicles lacking apparent connection to the chloroplast peripheral membrane (Figures 5A,D,F). The large invaginations could either be apparently totally enclosed within the chloroplast (Figures 5A,C,E,F), or still obviously connected to the cytoplasm (Figures 5B,E). In some instances spheroidal microbodies with a paracrystalline appearance were observed in close proximity to invaginated chloroplasts (Figure 5A); these may represent peroxisomes. Osmiophilic globules or plastoglobules were frequently observed close to abnormal chloroplasts (Figures 5E,F). In rare instances, chloroplasts with significant long terminal extensions resembling stromules were observed (Figure 5D). Aggregates of virions were occasionally observed near chloroplasts with large cytoplasmic inclusions, and rarely inside apparently totally enclosed invaginations (Figure 5E).

### Sequence variants of TGB3 in natural AltMV isolates

**Figure 6:**
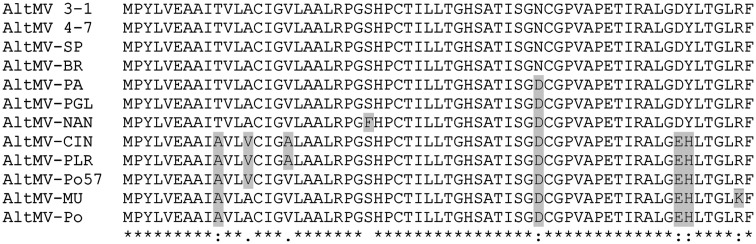
**Alignment of TGB3 amino acid sequences of different AltMV isolates**. Residues that differ from the consensus of phlox-derived isolates are highlighted; AltMV isolates CIN, PLR, Po57, MU, and Po are “portulaca-type” isolates that are also differentiated from “phlox-type” isolates by differences in the CP amino acid sequence (J. Hammond and M. Reinsel, unpublished data). The GenBank accession numbers of the isolates are: AltMV 3–1, GQ179646; AltMV 4–7, GQ179647; AltMV-SP, AY850931; AltMV-BR, AY850628; AltMV-PA, AY863024; AltMV-PGL, JQ405265; AltMV-NAN, JQ405267; AltMV-CIN, JQ405268; AltMV-PLR, JQ405266; AltMV-Po57, JQ405269; AltMV-MU, FJ822136; AltMV-Po, AY850930.

**Figure 7:**
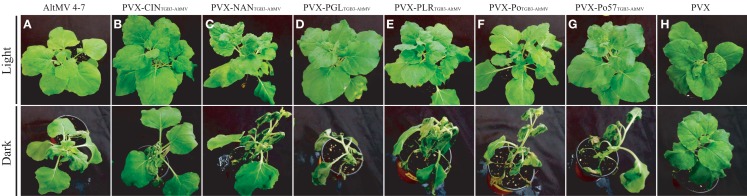
**Effect of growth in the dark on symptom severity in plants over-expressing AltMV TGB3 variants from a PVX vector**. Starting at 7 days after inoculation, *N. benthamiana* plants infected with AltMV 4–7, WT PVX, or PVX(TGB3 AltMV+) variants were incubated for 6 days under either diurnal light or constant dark conditions. Upper row – plants grown in light; Lower row – plants grown in dark. **(A)** AltMV 4–7 (positive control). **(B)** PVX-CIN_TGB3-AltMV_. **(C)** PVX-NAN_TGB3-AltMV_. (**D**) PVX-PGL_TGB3-AltMV_. **(E)** PVX-PLR_TGB3-AltMV_. **(F)** PVX-Po_TGB3-AltMV_. **(G)** PVX-Po57_TGB3-AltMV_. **(H)** WT PVX (negative control). Note increased severity of symptoms in dark-grown plants over-expressing AltMV TGB3 variants, with collapse of apical leaves, whereas AltMV 4–7 **(A)** shows slightly more severe symptoms, and WT PVX **(H)** shows minimal increase in symptom severity compared to light-grown plants.

**Figure 8:**
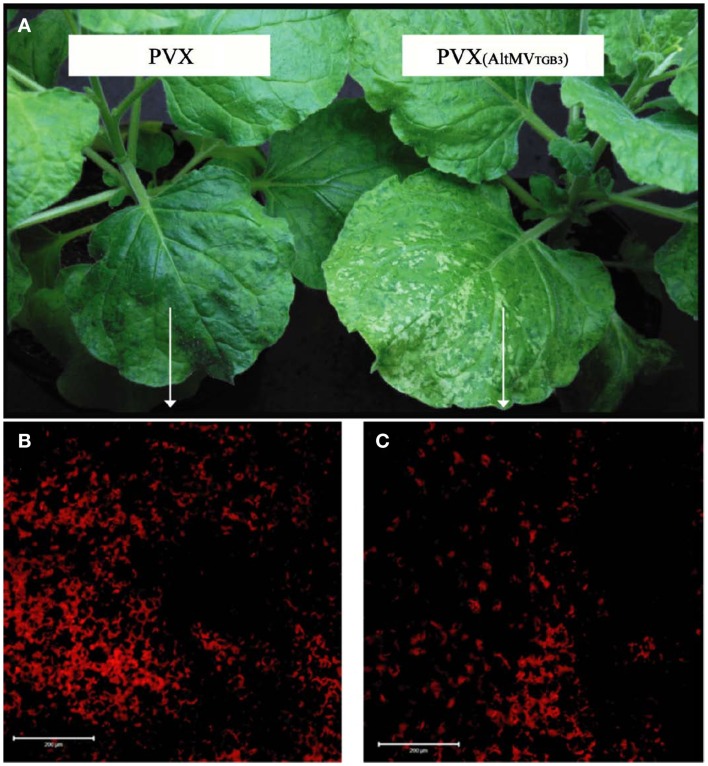
**Reduced chloroplast survival in plants with AltMV TGB3 over-expressed from the PVX genome**. Plants of *N. benthamiana* were inoculated with WT PVX, or PVX over-expressing AltMV TGB3, and grown under normal diurnal lighting. **(A)** Mild mosaic symptoms of WT PVX (left) and more severe symptoms including veinal necrosis induced by PVX over-expressing AltMV TGB3 (right). **(B)** LSCM visualization of chloroplast autofluorescence in the mesophyll layer of indicated area of leaf infected with PVX. **(C)** Significantly reduced chloroplast autofluorescence in indicated region of leaf infected with PVX over-expressing AltMV TGB3. Scale bar = 200 μm.

We have sequenced the TGB3 region of a number of AltMV isolates from various hosts (Hammond et al., 2006a,b; Lim et al., 2010b; J. Hammond and M. D. Reinsel, unpublished data) and the full sequence of a European portulaca isolate is also available (Ivanov et al., 2011). There are multiple TGB3 amino acid differences between these isolates (Figure 6), so we expressed each variant TGB3 sequence as an added gene from a PVX vector as previously described (Lim et al., 2010a) in *N. benthamiana*. Plants infected with AltMV 4–7 (Lim et al., 2010b), WT PVX, or PVX separately expressing each TGB3 variant were transferred at 7 dpi to be grown in either light (16 h/8 h diurnal cycle) or constant dark conditions for six further days. Plants infected with AltMV showed somewhat more severe symptoms after dark growth than in light (Figure 7A), while plants infected with PVX over-expressing TGB3 variants grown under dark conditions showed significantly more severe symptoms than plants maintained in the light (Figures 7B–G); plants infected with WT PVX showed milder, similar symptoms than AltMV-infected plants under both light and dark conditions (Figure 7H). As previously noted (Lim et al., 2010a) with plants infected with PVX(TGB3 AltMV+) and grown under normal light conditions, more severe symptoms including veinal-associated necrosis occurred (Figure 8A). Significantly fewer chloroplasts were observed by confocal microscopy in plants in which AltMV TGB3 variants were over-expressed, compared to plants infected with PVX (Figures 8B,C); the difference was greater for plants grown in the dark, but no correlation with specific amino acid substitutions was obvious (data not shown).

Comparison of the TGB3 sequences of different AltMV isolates revealed that AltMV-CIN (from cineraria) and AltMV-PLR (from hybrid annual phlox) both have an alanine residue at position 17 (Figure 6). This is of interest because mutant TGB3_VL(17,18)AR_ (see Figure 2) failed to accumulate at the chloroplast (Lim et al., 2010a), and failed to interact detectably with PsbO in BiFC (Figure 3). The occurrence of A17 in these two isolates suggests that it is L18 that is critical to chloroplast localization.

## Discussion

### AltMV TGB3 localization

*Alternanthera mosaic virus* TGB3 is a multifunctional protein, associated with both intra- and intercellular local movement, and with systemic movement; an infectious clone unable to produce TGB3 as a result of a premature stop codon is able to replicate and spread to a few adjacent epidermal cells, but not to move to the mesophyll (Lim et al., 2010a). TGB3 is therefore not absolutely required for replication, but the limited epidermal movement distinguishes the TGB3 mutant from clones unable to express either TGB2, or CP, which were unable to spread beyond the initially infected cell (Lim et al., 2010a), as previously noted for similar mutants of *White clover mosaic virus*, PVX, and BaMV (Beck et al., 1991; Lough et al., 2000; Lin et al., 2006). It has yet to be demonstrated whether AltMV TGB2 and TGB3 interact, as has been demonstrated with those of PVX (Samuels et al., 2007), BaMV (Lee et al., 2010), and some other TGB-expressing viruses (e.g., Solovyev et al., 2000; Cowan et al., 2002; Lim et al., 2008). PVX TGB3 has been shown to co-localize with the viral replicase at the ER in membrane-bound structures (Bamunusinghe et al., 2009), while AltMV TGB3 localizes to chloroplast membranes which may be the main site of AltMV replication, as the chloroplast membrane is the preferential site of virus accumulation (Lim et al., 2010a).

Whereas PVX TGB3 has been shown to localize to granular vesicles that also contain TGB2 (Schepetilnikov et al., 2005; Samuels et al., 2007; Ju et al., 2008) and with replicase at spherical bodies along the ER (Bamunusinghe et al., 2009), both N- and C-terminal AltMV TGB3 fluorescent fusion proteins localized to the chloroplast, and localization was not affected by co-expression of free TGB2, TGB3, or TGB2 + TGB3 (Lim et al., 2010a). Similarly, no TGB3 self-interaction was detected in BiFC (this study); thus unlike BaMV TGB3 (Lee et al., 2010), there is as yet no evidence for AltMV TGB3 self-interaction. Agroinfiltrated PVX GFP:TGB3 and DsRed:TGB3 were localized primarily at the periphery of epidermal cells, while AltMV TGB3 fusions were observed almost exclusively in the mesophyll in association with the chloroplasts when agroinfiltrated under the same conditions (Lim et al., 2010a). It is the N-terminal domain of AltMV TGB3 that is critical for chloroplast targeting, even when fused downstream of GFP or DsRed. Mutation of TGB3 residues VL(17,18)AR was sufficient to ablate chloroplast targeting and allow accumulation at the periphery of epidermal cells, and in the context of an infectious clone yielded a virus unable to move beyond the epidermal layer (Lim et al., 2010a). There was therefore strong evidence of a link between TGB3 and the chloroplast, which is necessary for systemic movement of AltMV.

The chloroplast association was further demonstrated by over-expression of AltMV TGB3 from either AltMV or PVX; plants infected with either virus over-expressing TGB3 developed more severe symptoms, including veinal necrosis. In *N. tabacum*, a non-host of AltMV, PVX over-expressing AltMV TGB3 induced necrotic local lesions rather than the chlorotic local lesions induced by WT PVX, whereas PVX TGB3 over-expressed from AltMV in *N. benthamiana* did not increase symptom severity (Lim et al., 2010a). Chloroplast invaginations similar to those reported with plants infected by TYMV (Ushiyama and Matthews, 1970) were observed in *N. benthamiana* infected with either AltMV or PVX over-expressing AltMV TGB3, and fewer intact chloroplasts were observed in plants infected with PVX over-expressing AltMV TGB3 than in controls infected with WT PVX (Lim et al., 2010a; and this study); the observed chloroplast destruction mirrors that reported for TYMV under normal light conditions by Chalcroft and Matthews (1966).

### PsbO localization and functions

It was therefore of considerable interest when we identified an interaction between AltMV TGB3 and AtPsbO1 by screening an *A. thaliana* cDNA library by the Y2H method, as PsbO is a nuclear-encoded major component of the chloroplast-localized OEC of PS II (Tyagi et al., 1987). We also cloned a PsbO gene from *N. benthamiana*, based on the sequence determined by Sui et al. (2006), and confirmed interaction between TGB3 and both AtPsbO1 and NbPsbO by BiFC; the BiFC results clearly demonstrate co-localization of TGB3 and PsbO surrounding chloroplasts in mesophyll cells.

The chloroplast-localized interaction of TGB3 and PsbO observed by BiFC was confirmed by the co-localization of DsRed:TGB3 with GFP:PsbO; interestingly the siting of the interaction between appressed chloroplasts is similar to the observation of chloroplast clumping caused by TYMV infection (Chalcroft and Matthews, 1966), although in the current instance in the absence of viral infection and other viral proteins. As DsRed:TGB3 expressed alone induced punctuate spots, whereas GFP:PsbO expressed alone accumulated surrounding the chloroplasts, perhaps the interaction draws the chloroplasts together and creates a more favorable environment for replication complexes to be protected from host defenses.

While Sui et al. (2006) were aware that *N. tabacum* has multiple copies of the *psbO* gene, only one copy was cloned from *N. benthamiana* until Pérez-Bueno et al. (2011) cloned four isoforms and demonstrated that only three amino acids differ between the mature forms of NbPsbO1 and NbPsbO2; two further residues differ between the 85-residue signal peptides of these isoforms. The NbPsbO of Sui et al. (2006) is identical to NbPsbO2 (Pérez-Bueno et al., 2011) except for the third residue of the signal peptide (Ala, Val, and Thr in NbPsbO, NbPsbO1, and NbPsbO2, respectively), as a consequence of the NbPsbO PCR primer designed on the basis of *N. tabacum* PsbO (Sui et al., 2006); our construct is therefore essentially equivalent to NbPsbO2. NbPsbO1 and NbPsbO2 are presumed to have different functionality than NbPsbO3 and NbPsbO4 (Pérez-Bueno et al., 2011), as reported for AtPsbO1 and AtPsbO2 in *Arabidopsis* due to three specific amino acid differences in the C-terminal domain (Murakami et al., 2005; Lundin et al., 2007a,b). In *Arabidopsis*, PsbO2 has threefold higher GTPase activity that PsbO1, whereas PsbO1 is expressed at higher levels and supports PS II activity better under high light conditions; AtPsbO2 is also known to regulate dephosphorylation and turnover of PS II reaction center D1 protein (Lundin et al., 2007a, 2008). However, comparison of *Arabidopsis* and NbPsbO amino acid sequences shows multiple differences between both of the *Arabidopsis* proteins and all of the *N. benthamiana* homologs (Pérez-Bueno et al., 2011), such that functional differences between NbPsbO isoforms cannot be readily predicted from the sequences. Further work with different isoforms of NbPsbO will be necessary to identify possible differences in interaction with AltMV TGB3. It is possible that TGB3 may interact with all isoforms; there are limited amino acid differences between isoforms (95–96% identity), although some of these differences may affect binding between PsbO and PsbP, or GTP binding (Pérez-Bueno et al., 2011). Only one GTP motif residue, in motif G1, differentiates NbPsbO2 from the other isoforms, whereas the G2/G3 motif is identical in all isoforms, and the G4 motif is GKPE in NbPsbO1/2, and GKPD in NbPsbO3/4, as in AtPsbO1 and AtPsbO2, respectively.

The extrinsic proteins of PS II have recently been reviewed by Bricker et al. (2012): PsbO binds GTP with high affinity and functions as a GTPase; in this role it may control the phosphorylation state of D1 (the chloroplast-encoded core protein of PS II), which is coupled to efficient PS II cycling. PsbO may therefore impose important regulatory controls on photosynthesis; different isoforms may exert differential function, and depletion of one PsbO isoform may have a different effect than depletion of another. In *Arabidopsis*, PsbO1 primarily supports normal oxygen evolution, while PsbO2 regulates the phosphorylation state and turnover of D1; AtPsbO2 has substantially higher GTPase activity, while functioning poorly in support of oxygen evolution. PsbO is also generally assumed to be required for PsbP binding, while PsbP is required for the association of PsbQ to PS II. The chloroplast-encoded PS II intrinsic core protein D1 is light-regulated, and translation is controlled by signals initiated by both PS I and PS II (Trebitsh and Danon, 2001).

PsbO is nuclear-encoded, and is directed to the chloroplast by an 85-residue signal peptide which is cleaved from the mature protein prior to localization of PsbO in the thylakoid lumen. Within the thylakoid lumen, PsbO is proposed to stabilize the dimeric structure of the PS II complex, and also to bind and hydrolyze GTP. PsbO is known to dissociate from its docking site upon photoinactivation of PS II electron transport, and can be released under non-inhibitory light conditions as well as at pH 6.0 in darkness; GTP stimulates light-induced release of PsbO from inactivated PS II complexes, resulting in degradation of the PS II reaction center protein D1. GTP binding and hydrolysis occur readily in darkness, potentially releasing PsbO from the luminal surface of PS II (see Lundin et al., 2007b). The D1 protein is located in the thylakoid stromal membrane, and rapid turnover of dissociated D1 requires the incorporation of freshly synthesized D1 to rebuild PS II. PsbO is thus an important regulator of D1 protein turnover (Lundin et al., 2008), and is the minimal and most crucial luminal extrinsic component for an adequate function of water oxidation to molecular oxygen in PS II (Lundin et al., 2007b).

### Causes and effects of chloroplast damage

In the current study we over-expressed multiple natural variants of AltMV TGB3 from PVX, and examined chloroplasts in leaves of plants grown either under diurnal lighting, or in continuous darkness. Whereas PVX-infected controls showed little difference between light- and dark-grown plants, all plants over-expressing AltMV TGB3 variants showed more severe symptoms than WT PVX in light conditions, and additionally caused significant chloroplast damage and tissue collapse under dark conditions. One possible explanation for this observation is that cytoplasmic interaction of TGB3 and PsbO interferes with the recruitment of fresh PsbO to the chloroplast and PS II, affecting turnover of D1, further destabilizing the thylakoids and PS II, and leading to subsequent chloroplast disruption. Neither infection by WT PVX under either light or dark conditions, nor the over-expression of TGB3 under light conditions, is sufficient to cause severe symptoms or major chloroplast damage. The lack of light-induced expression of D1 when plants were grown in the dark, in combination with inhibition of PsbO recruitment to the thylakoids, is the probable cause of the observed severe symptoms and chloroplast destruction.

Further investigation is needed to examine the effects of these treatments on relative levels of different chloroplast proteins, and in particular on the ratios of PsbO to PsbP and PsbQ, and to D1. Differential effects on components of the PS II complex have been reported with other viruses, at least partially correlating with tobamovirus and cucumovirus symptom severity in *N. tabacum* and *N. benthamiana* (Takahashi et al., 1991; Takahashi and Ehara, 1992; Rahoutei et al., 2000; Pérez-Bueno et al., 2004; Sui et al., 2006); however, all components of the PS II complex were depleted in *N. tabacum* infected with the *flavum* (yellowing) strain of TMV (Lehto et al., 2003). Interestingly, silencing of *psbO* resulted in a 10-fold increase in TMV accumulation, whereas infection with TMV normally down-regulated PsbO mRNA levels suggesting the possibility that inhibition of the OEC and PS II optimizes conditions for infection by suppressing basal plant defense mechanisms (Abbink et al., 2002). It will be of interest to see whether VIGS of *psbO* will increase accumulation of AltMV as observed for TMV (Abbink et al., 2002), or decrease accumulation as reported with PVX when plastocyanin expression was reduced (Qiao et al., 2009), or for BaMV when *cPGK* was silenced (Lin et al., 2007).

Although over-expression of AltMV TGB3 clearly has a significant effect on chloroplast survival (causing veinal necrosis and reduced chloroplast numbers, especially under dark conditions), the mechanism is not clear. Damage to the chloroplasts may suppress the plant’s basal defense mechanisms, allowing the virus to replicate unhindered; however, overall levels of virus replication appear little altered, as replication of neither AltMV nor PVX over-expressing AltMV TGB3 was obviously enhanced (Lim et al., 2010a). Whereas we have established a clear interaction between PsbO and TGB3, and demonstrated that the N-terminal domain of TGB3 is required for the interaction, we have yet to determine which domains of PsbO are involved, and exactly where the interaction occurs, as visualization at the chloroplast does not preclude PsbO from acting to transport TGB3 to the chloroplast. The N-terminal domain of TGB3 contains signals required for chloroplast localization, and mutation of VL(17,18)AR is sufficient to prevent both chloroplast attachment and to restrict movement of otherwise infectious AltMV to a few cells within the epidermis (Lim et al., 2010a), as well as essentially abolishing the BiFC interaction with PsbO (this work). It should be noted that the N-terminal domain of TGB3 containing the chloroplast localization sequence is highly constrained as it overlaps with the C-terminus of TGB2 in a different reading frame, and that the TGB3_VL(17,18)AR_ mutant maintains the WT TGB2 amino acid sequence (Lim et al., 2010a). Because TGB3_VL(17,18)AR_ neither localizes to the chloroplast (Lim et al., 2010a), nor interacts with PsbO, we were unable to distinguish between the possibilities that chloroplast localization of TGB3 is required for interaction with PsbO, or that PsbO interaction is necessary for targeting of TGB3 to the chloroplast. In future work we will determine whether TGB3 interacts with the PsbO signal peptide or with the mature protein.

### Potential PsbO interaction domains

Alignment of AtPsbO and NbPsbO variants shows that there are many differences within the signal peptide domain, except for 15 fully conserved residues immediately upstream of the cleavage site, whereas there is a high degree of identity throughout the mature PsbO peptide (Pérez-Bueno et al., 2011; and data not shown). It is therefore most likely that TGB3 interacts with the functional portion of PsbO rather than the signal peptide, although the localization of the interaction is not yet known. In contrast, it has been demonstrated that PVX CP interacts specifically with the transit peptide of plastocyanin (Qiao et al., 2009), a nuclear-encoded chloroplast protein involved in PS I and accumulating in the thylakoid lumen (Lawrence and Kindle, 1997); plastocyanin precursor protein may therefore target PVX CP to the chloroplast, but whether plastocyanin is also sufficient to act as a carrier to transport CP into the organelle is not clear (Qiao et al., 2009). Our evidence to date suggests that TGB3 remains outside the chloroplast membrane (Lim et al., 2010a). As TGB3 does not have a canonical signal sequence, it is possible that interaction of TGB3 with PsbO results in transport of TGB3 to the chloroplast, where electron microscopy suggests that invaginations result from TGB3 insertion forming protrusions into, rather than across, the chloroplast membrane (Lim et al., 2010a). It is notable that BaMV TGB3 has been shown to localize to curved domains of the cortical ER (Lee et al., 2010), and that a sorting signal critical for targeting of BaMV TGB3 to punctae within curved ER tubules has been identified; however, while BaMV TGB3 targets curved domains of the ER, it is unable to shape the ER (Wu et al., 2011).

### Functions of viral:Chloroplast interactions

The TGB proteins are often considered to interact with each other in order to transport viral RNA between cells via the plasmodesmata, supported by the ability to exchange the complete TGB to produce functional hybrid viruses, and multiple reports of co-localization of TGB2 and TGB3 (e.g., Solovyev et al., 2000; Morozov and Solovyev, 2003; Verchot-Lubicz, 2005; Samuels et al., 2007). TGB3 and the replicase of PVX have been shown to co-localize in membrane-bound spherical bodies including the ER marker BiP, at an early stage of infection (Bamunusinghe et al., 2009). More recently, PVX TGB1 has been demonstrated to reorganize actin and endomenbranes into the X-body, which was also shown to include CP, granular vesicles containing TGB2 and TGB3, and non-encapsidated viral RNA (Tilsner et al., 2012). While the presence of the replicase itself was not directly demonstrated, the TGB2/TGB3 granular vesicles have previously been associated with replicase and ribosomes (Ju et al., 2005; Bamunusinghe et al., 2009), and the X-body is presumed to be the viral replication “factory” (Tilsner et al., 2012). Whereas Golgi bodies were found within the X-bodies (Tilsner et al., 2012), the inclusion of chloroplasts was not noted. Yan et al. (2012) further examined aggregates of PVX TGB1/TGB2/TGB3, confirming the close association of the TGB proteins, without any chloroplast association. Chloroplasts were also not obviously associated with perinuclear ER-derived membrane aggregations in cells infected with the comovirus *Cowpea mosaic virus* (CPMV; Carette et al., 2000) or the nepovirus *Grapevine fanleaf virus* (GFLV; Ritzenthaler et al., 2002).

In contrast to the situation with PVX, CPMV, and GFLV, the potyvirus TuMV has been shown to recruit ER membranes and chloroplasts sequentially through the action of 6K2-containing membranous vesicles, which aggregate and induce invaginations at the chloroplast membrane (Wei et al., 2010). Further examination of this system revealed perinuclear globular structures that included ER, Golgi bodies, COPII coatamers, and chloroplasts as well as viral proteins (Grangeon et al., 2012).

Manfre et al. (2011) summarize a number of studies suggesting that the chloroplast plays an important cellular role during viral invasion, which might include the location of viral replication, or activity in host defenses. As AltMV TGB3 is part of the viral movement complex, and AltMV replication is associated with the chloroplast (Lim et al., 2010a), both of these roles may be relevant. A number of viruses have been shown to repress expression of multiple nuclear-encoded chloroplast proteins (Dardick, 2007; Shimizu et al., 2007; Yang et al., 2007), and multiple chloroplast proteins interact with various potyviral proteins in Y2H screens, indicating that many viruses disrupt or modify chloroplast structure or function while establishing infection (Manfre et al., 2011). Whether such interactions interfere with host defenses to promote systemic susceptibility is still unclear, but it has been proposed that the chloroplast plays a critical role in host defense (Genoud et al., 2002; Griebel and Zeier, 2008) and that viral interactions with the chloroplast may interfere in defense signaling (Abbink et al., 2002; Lehto et al., 2003). Manfre et al. (2011) showed that silencing of several individual photosynthetic proteins led to increases in numbers of TuMV infection foci compared to controls, suggesting that a general effect on photosynthetic capacity or chloroplast function influences host susceptibility; infections under low light also resulted in increased numbers of infection foci and increased rate of systemic movement. Treatment with the chloroplast protein synthesis inhibitor Lin increased both numbers of foci and rate of systemic movement even under light conditions, indicating that the photosynthetic or energy-production functions of the chloroplast are essential for plant defense mechanisms (Manfre et al., 2011). Although salicylic acid (SA)-mediated host defense mechanisms are light-dependent, and SA is thought to be synthesized in the chloroplast, no direct relationship could be demonstrated between SA and numbers of TuMV infection foci; an alternative hypothesis that light and chloroplast function influence the ability of viruses to establish replication centers was considered (Manfre et al., 2011). TuMV has indeed recently been shown to establish replication complexes at the outer membrane of the chloroplast (Wei et al., 2010), as AltMV is also believed to do, in part through the interaction of TGB3 at the chloroplast (Lim et al., 2010a). The interaction of TGB3 with PsbO may thus both interfere with the host basal defenses, and establish the location for the AltMV replication complex at the chloroplast surface.

### Differences between AltMV and PVX

*Alternanthera mosaic virus* is a member of the genus *Potexvirus*, yet has several clear differences from the type member, PVX. AltMV TGB3 agroinfiltrated alone is targeted to the mesophyll and specifically to the chloroplast (Lim et al., 2010a), whereas PVX TGB3 is targeted to the ER (Ju et al., 2008), and accumulates primarily in the epidermis (Lim et al., 2010a). Fluorescence *in situ* hybridization to AltMV-infected leaf sections revealed that AltMV RNA was primarily associated with chloroplasts in the mesophyll, with little signal from either epidermis (Lim et al., 2010a), whereas PVX has no obvious reported association with chloroplasts. No beaded sheets of TGB1 are readily discernible in AltMV-infected tissue, whereas the beaded sheets of PVX TGB1 are characteristic and easily detected (e.g., Davies et al., 1993). In contrast, paracrystalline inclusions are frequently observed in both the nucleus and cytoplasm of AltMV-infected cells (J. Hammond, H.-S. Lim, and M. M. Dienelt, unpublished data) and these may represent aggregates of TGB1; GFP-TGB1 aggregates in both the cytoplasm and nucleus (Lim et al., 2010c). Further work will be required to determine whether AltMV replication complexes are indeed associated with the chloroplast rather than the nucleus (as for TYMV; Prod’Homme et al., 2001, 2003), or incorporate chloroplasts in association with the nucleus (as for TuMV; Grangeon et al., 2012). Considering the differences in subcellular localization of TGB3, in TGB2/TGB3 interactions, in TGB1 subcellular organization, and apparent sites of replication, there is much to be learned by further comparison of AltMV and PVX. It will also be of interest to further examine the host proteins interacting with the respective viral proteins, to determine the common features and further differences between these two members of the genus *Potexvirus*.

## Conflict of Interest Statement

The authors declare that the research was conducted in the absence of any commercial or financial relationships that could be construed as a potential conflict of interest.
